# Expression mRNA pattern of retinoic acid and retinoid X nuclear receptor subtypes in thyroid carcinomas

**DOI:** 10.1186/1756-6614-6-S2-A11

**Published:** 2013-04-05

**Authors:** Julius Brtko, Dana Macejova, Stefan Galbavy, Lucia Bialesová, Jan Podoba

**Affiliations:** 1 Institute of Experimental Endocrinology, Slovak Academy of Sciences, Bratislava, Slovak Republic; 2 Saint Elizabeth Institute of Oncology, Bratislava, Slovak Republic

## 

Retinoid receptors (RARs) upon a proper ligand binding act as all-trans retinoic acid-inducible transcription factors interacting as heterodimers with retinoid X receptors (rexinoid receptors, RXRs).

The objective of this study was to evaluate all retinoid/rexinoid nuclear receptor subtypes (RARalpha, RARbeta, RARgamma, RXRalpha, RXRbeta, RXRgamma) expression pattern in thyroid tumour tissue of patients with different types of thyroid cancer in order to compare it with that of the intact thyroid tissue of the corresponding patient. The expression of the retinoid/rexinoid nuclear receptor subtypes has been analyzed by the semiquantitative RT-PCR technique.

Papillary thyroid carcinoma (PTC) patients expressed RXRgamma when compared to non-neoplastic thyroid tissues of the corresponding patients that were lacking to express RXRgamma or its expression was very low. Moreover, we have found significantly increased expression of RARalpha and RARgamma in overall group of PTC patients. This increase was detected in cases with positive lymph node metastasis (LNM), but not with negative LNM. On the other hand, RARbeta was significantly reduced in the subgroup of classic variant (CV) of papillary carcinoma.

Expression of RXRgamma in the patient with anaplastic carcinoma was found to be lower than that of patients with papillary carcinoma. Follicular adenoma or malignant lymphoma, and also nonmalignant follicular nodules were expressing all RAR and RXR subtypes. On the other hand, hyperplastic nodule was found to express all RAR or RXR subtypes, except RARgamma.

In conclusion, the data on the differences in RAR and RXR subtype mRNA expression patterns in various thyroid carcinomas might thus enhance therapeutical potentialities, and thus they may find exploitation in clinical oncology, predominantly, in the differential diagnostics of human thyroid neoplasms.

